# Supplementary material for: H4K16ac activates the transcription of transposable elements and contributes to their cis-regulatory function
Source: Nat Struct Mol Biol. 2023 Jun 12;30(7):935–47. doi: 10.1038/s41594-023-01016-5 (PMC10352135; doi:10.1038/s41594-023-01016-5)
Supplement: Supplementary file 1 — Supplementary Fig. 1 and Tables 1–5. [file 41594_2023_1016_MOESM1_ESM.pdf]

nature structural & molecular biology

Article https://doi.org/10.1038/s41594-023-01016-5

H4K16ac activates the transcription of transposable elements and contributes to their *cis*-regulatory function

In the format provided by the
authors and unedited

This Supplementary file includes:

Supplementary Figure 1
Supplementary Tables 1 to 5

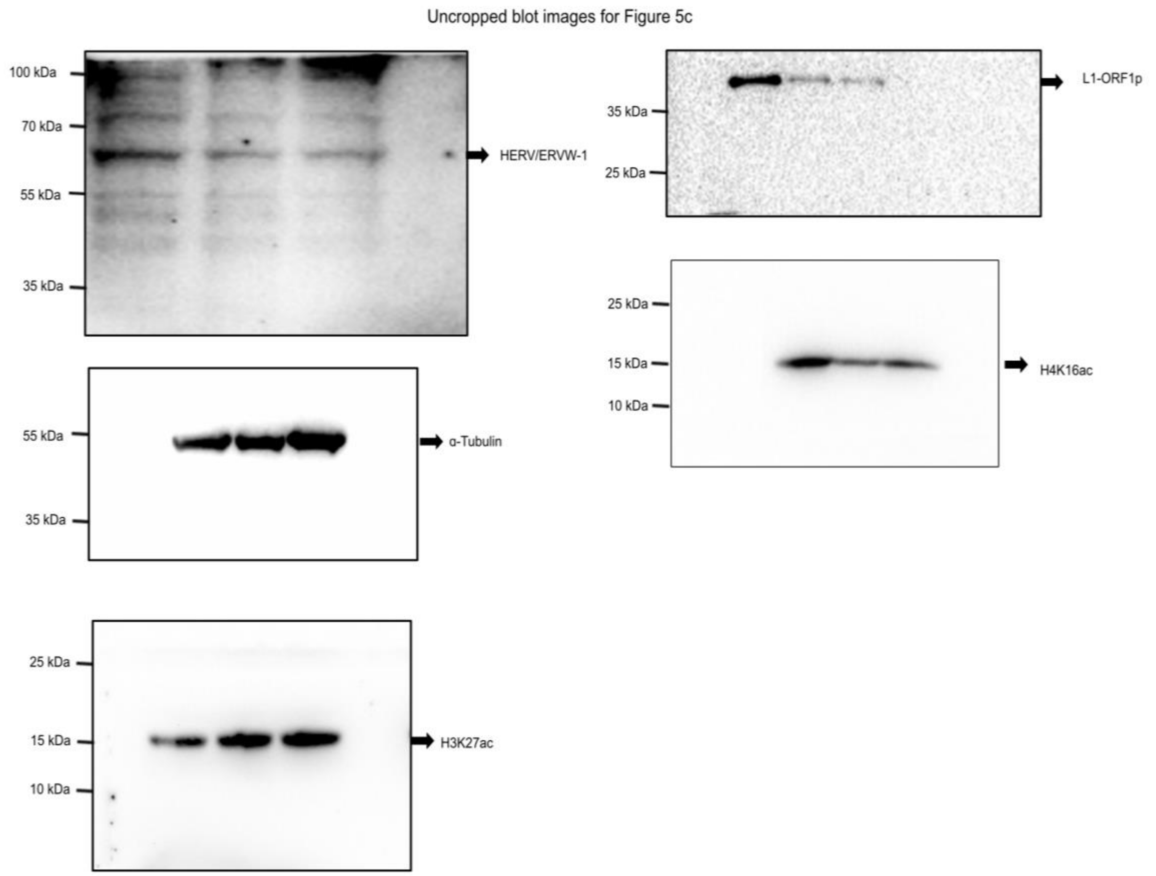

Supplementary Figure 1: Uncropped western blotting images for Figure 5c

Supplementary Table 1. Data generated and analysed in this study

	Experiment	GEO ID	Cell line, antibody, replicate number	Reference
1	CUT&Tag	GSM6043377	H9, H3K27ac Rep1	This study
2	CUT&Tag	GSM6043378	H9, H3K27ac Rep2	This study
3	CUT&Tag	GSM6043379	H9, H3K27ac Rep3	This study
4	CUT&Tag	GSM6043380	H9, H3K27me3 Rep1	This study
5	CUT&Tag	GSM6043381	H9, H3K27me3 Rep2	This study
6	CUT&Tag	GSM6043382	H9, H3K36me3 Rep1	This study
7	CUT&Tag	GSM6043383	H9, H3K4me1 Rep1	This study
8	CUT&Tag	GSM6043384	H9, H3K4me1 Rep2	This study
9	CUT&Tag	GSM6043385	H9, H3K4me1 Rep3	This study
10	CUT&Tag	GSM6043386	H9, H3K4me3 Rep1	This study
11	CUT&Tag	GSM6043387	H9, H3K4me3 Rep2	This study
12	CUT&Tag	GSM6043388	H9, H3K4me3 Rep3	This study
13	CUT&Tag	GSM6043389	H9, H4K12ac Rep1	This study
14	CUT&Tag	GSM6043390	H9, H4K12ac Rep2	This study
15	CUT&Tag	GSM6043391	H9, H4K12ac Rep3	This study
16	CUT&Tag	GSM6043392	H9, H4K16ac Rep1	This study
17	CUT&Tag	GSM6043393	H9, H4K16ac Rep2	This study
18	CUT&Tag	GSM6043394	H9, H4K16ac Rep3	This study
19	CUT&Tag	GSM6043395	H9, IgG Rep1	This study
20	CUT&Tag	GSM6043396	H9, IgG Rep2	This study
21	CUT&Tag	GSM6043397	H9, IgG Rep3	This study
22	CUT&Tag	GSM6043398	H9, H3K122ac Rep1	This study

23	CUT&Tag	GSM6043399	H9, H3K122ac Rep2	This study
24	CUT&Tag	GSM6043400	H9, H3K9me3 Rep1	This study
25	CUT&Tag	GSM6043401	H9, H3K9me3 Rep2	This study
26	CUT&Tag	GSM6043402	TDF, icas9 Igg	This study
27	CUT&Tag	GSM6043403	TDF, icas9 H4K16ac Rep1	This study
28	CUT&Tag	GSM6043404	TDF, icas9 H3K27ac	This study
29	CUT&Tag	GSM6043405	TDF, icas9_K9me3	This study
30	CUT&Tag	GSM6043406	TDF, MSL3KO_Igg	This study
31	CUT&Tag	GSM6043407	TDF, MSL3KO H4K16ac	This study
32	CUT&Tag	GSM6043408	TDF, MSL3KO H3K27ac	This study
33	CUT&Tag	GSM6043409	TDF, MSL3KO H3K9me3	This study
34	CUT&Tag	GSM6043410	TDF, MSL1KO Igg	This study
35	CUT&Tag	GSM6043411	TDF, icas9 H4K16ac Rep2	This study
36	CUT&Tag	GSM6043412	TDF, icas9 H4K16ac Rep3	This study
37	CUT&Tag	GSM6043413	TDF, MSL1KO H4K16ac Rep1	This study
38	CUT&Tag	GSM6043414	TDF, MSL1KO H4K16ac Rep2	This study
39	CUT&Tag	GSM6043415	HEK, IgG Rep1	This study
40	CUT&Tag	GSM6043416	HEK, H4K16ac Rep1	This study
41	CUT&Tag	GSM6043417	HEK, H4K16ac Rep2	This study
42	CUT&Tag	GSM6043418	HEK, H3K27ac Rep1	This study
43	CUT&Tag	GSM6043419	HEK, H3K27ac Rep2	This study
44	CUT&Tag	GSM6043420	HeLa, IgG Rep1	This study
45	CUT&Tag	GSM6043421	HeLa, H4K16ac Rep1	This study
46	CUT&Tag	GSM6043422	HeLa, H4K16ac Rep2	This study

47	CUT&Tag	GSM6043423	HeLa, H3K27ac Rep1	This study
48	CUT&Tag	GSM6043424	HeLa, H3K27ac Rep2	This study
49	CUT&Tag	GSM6043425	K562, IgG Rep1	This study
50	CUT&Tag	GSM6043426	K562, H4K16ac Rep1	This study
51	CUT&Tag	GSM6043427	K562, H4K16ac Rep2	This study
52	CUT&Tag	GSM6043428	K562, H3K27ac Rep1	This study
53	CUT&Tag	GSM6043429	K562, H3K27ac Rep2	This study
54	CUT&Tag	GSM6043430	LNCaP, IgG Rep1	This study
55	CUT&Tag	GSM6043431	LNCaP, H4K16ac Rep1	This study
56	CUT&Tag	GSM6043432	LNCaP, H3K27ac Rep1	This study
57	CUT&Tag	GSM6043433	LNCaP, H3K27ac Rep2	This study
58	CUT&Tag	GSM6043434	PC3, IgG Rep1	This study
59	CUT&Tag	GSM6043435	PC3, H4K16ac Rep1	This study
60	CUT&Tag	GSM6043436	PC3, H4K16ac Rep2	This study
61	CUT&Tag	GSM6043437	PC3, H3K27ac Rep1	This study
62	CUT&Tag	GSM6043438	PC3, H3K27ac Rep2	This study
63	CUT&Tag	GSM6043439	RWPE, IgG Rep1	This study
64	CUT&Tag	GSM6043440	RWPE, H4K16ac Rep1	This study
65	CUT&Tag	GSM6043441	RWPE, H3K27ac Rep1	This study
66	CUT&Tag	GSM6043442	SH-SY5Y, H4K16ac Rep1	This study
67	CUT&Tag	GSM6043443	SH-SY5Y, H4K16ac Rep2	This study
68	CUT&Tag	GSM6043444	SH-SY5Y, H3K27ac Rep1	This study
69	CUT&Tag	GSM6043445	SH-SY5Y, H3K27ac Rep2	This study

70	ATAC-seq	GSM6043367	H9, Scr ATAC Rep1	This study
71	ATAC-seq	GSM6043368	H9, Scr ATAC Rep2	This study
72	ATAC-seq	GSM6043369	H9, Scr ATAC Rep3	This study
73	ATAC-seq	GSM6043370	H9, MSL3 KD ATAC Rep1	This study
74	ATAC-seq	GSM6043371	H9, MSL3 KD ATAC Rep2	This study
75	ATAC-seq	GSM6043372	H9, MSL3 KD ATAC Rep3	This study
76	ATAC-seq	GSM6043373	TDF, icas9 ATAC Rep1	This study
77	ATAC-seq	GSM6043374	TDF, icas9 ATAC Rep2	This study
78	ATAC-seq	GSM6043375	TDF, MSL1 KO ATAC Rep1	This study
79	ATAC-seq	GSM6043376	TDF, MSL1 KO ATAC Rep2	This study
80	ATACseq	GSM4770996	THP1, sgNegCtrl ATAC	Radzisheuskaya 2021
81	ATACseq	GSM4770997	THP1, sgMSL1_KO ATAC	Radzisheuskaya 2021
82	RNA-seq	GSM6043446	H9, Scr RNA-seq Rep1	This study
83	RNA-seq	GSM6043447	H9, Scr RNA-seq Rep2	This study
84	RNA-seq	GSM6043448	H9, MSL3KD RNA-seq Rep1	This study
85	RNA-seq	GSM6043449	H9, MSL3KD RNA-seq Rep2	This study
86	Hi-C	GSM3262956	H9, HiC.Rep1	Zhang et al 2019
87	Hi-C	GSM3262957	H9, HiC.Rep2	Zhang et al 2019
88	ChIP-seq	GSM1358821	IMR90, H4K16ac Prol ChIP Rep1	Zhang et al 2019
89	ChIP-seq	GSM1358822	IMR90, H4K16ac Prolif ChIP Rep2	Zhang et al 2019
90	ChIP-seq	GSM1358823	IMR90, H4K16ac Sen ChIP Rep 1	Zhang et al 2019
91	ChIP-seq	GSM1358824	IMR90, H4K16ac Sen ChIP Rep 2	Zhang et al 2019

92	ChIPseq	GSE84618	Brain, H4K16ac-Input.AD.bw	Nativio 2018
93	ChIPseq	GSE84618	Brain, H4K16ac-Input.Old.bw	Nativio 2018
94	ChIPseq	GSE84618	Brain, H4K16ac-Input.Young.bw	Nativio 2018
95	RNA-seq	GSM4278153	TDF, WT Rep 1	Monserrat et al 2021
96	RNA-seq	GSM4278154	TDF, WT Rep 2	Monserrat et al 2021
97	RNA-seq	GSM4278155	TDF, WT Rep 3.	
98	RNA-seq	GSM4278141	TDF, MSL1 KO Rep 1	
99	RNA-seq	GSM4278141	TDF, MSL1 KO Rep 1	
100	RNA-seq	GSM4278142	TDF, MSL1 KO Rep 2	Monserrat et al 2021
101	RNA-seq	GSM4278143	TDF, MSL1 KO Rep 3	Monserrat et al 2021
102	RNA-seq	GSM4278147	TDF, MSL3 KO Rep 1	Monserrat et al 2021
103	RNA-seq	GSM4278148	TDF, MSL3 KO Rep 2	Monserrat et al 2021
104	RNA-seq	GSM4278149	TDF, MSL3 KO Rep 3	Monserrat et al 2021
105	ATAC-seq	GSM5219560	H9 hESC WT_ATAC-seq Rep1	Hsieh et al 2022
106	ATAC-seq	GSM5219561	H9 hESC WT_ATAC-seq Rep2	Hsieh et al 2022

Supplementary Table 2
Mapping details and number of peaks detected in all replicates across histone modifications.

Sample	Total read pairs (post trimming)	#unique aligned	#multi-aligned	unique aligned %age	multi-aligned %age	# of peaks
H9, H3K27ac Rep1	5267795	3461853	1177926	65.72	22.36	235572
H9, H3K27ac Rep2	24283828	17273549	5108095	71.13	21.03	332822
H9, H3K27ac Rep3	21041964	14397784	5081506	68.42	24.15	317466
H9, H3K27me3 Rep1	1059518	512357	158594	48.36	14.97	14832
H9, H3K27me3 Rep2	2330065	1682644	408995	72.21	17.55	4921
H9, H3K4me1 Rep1	8726469	6222892	1431697	71.31	16.41	173680
H9, H3K4me1 Rep2	16756188	12116934	3139439	72.31	18.74	158201
H9, H3K4me1 Rep3	35390485	27655989	6124551	78.15	17.31	141821
H9, H3K4me3 Rep1	6822949	5261704	791453	77.12	11.60	29657
H9, H3K4me3 Rep2	40759484	33771216	4452970	82.85	10.92	28240
H9, H3K4me3 Rep3	1452386	1152155	183540	79.33	12.64	24204
H9, H4K12ac Rep1	7805697	4827628	1887568	61.85	24.18	274100
H9, H4K12ac Rep2	23829711	15960547	5831918	66.98	24.47	345073
H9, H4K12ac Rep3	27318999	17788703	8024831	65.11	29.37	253197
H9, H4K16ac Rep1	8831804	5604427	2477020	63.46	28.05	288817
H9, H4K16ac Rep2	18212861	10648064	4116399	58.46	22.60	370833
H9, H4K16ac Rep3	21797848	13885613	6910663	63.70	31.70	256460
H9, H3K122ac Rep1	1434855	733254	532893	51.10	37.14	192177
H9, H3K122ac Rep2	2802275	1621322	1077241	57.86	38.44	251153
H9, H3K9me3 Rep1	16787273	6343184	10151605	37.79	60.47	43032
H9, H3K9me3 Rep2	1776891	380612	1239764	21.42	69.77	43921
H9, IgG Rep1	1455306	509592	397818	35.02	27.34	11089
H9, IgG Rep2	1000089	386107	316372	38.61	31.63	10193
H9, IgG Rep3	10973836	6075158	3832333	55.36	34.92	60305

Supplementary Table 3
Statistics for the histone modification reproducible peaks at genomic elements

A) Ratio of enrichment over background for genomic elements overlapping histone modification peaks

	Alu	L1	LTR	TSS	Genes
IgG	0.69	0.60	0.32	25.35	2.34
H3K122ac	0.60	**5.45**	0.76	**2.58**	1.09
H3K12ac	**2.70**	2.60	1.40	15.60	2.04
H4K16ac	**1.89**	**2.93**	**1.86**	**1.02**	1.31
H3K27ac	**2.21**	1.54	1.58	12.22	1.80
H3K27me3	0.48	0.08	0.32	11.83	1.68
H3K4me1	1.14	1.67	1.17	16.68	1.71
H3K4me3	0.86	1.57	0.39	63.32	2.78
H3K9me3	0.65	**19.18**	**1.98**	**0.46**	0.67

B) Percentage of genomic elements overlapping reproducible peaks of histone modifications

	% of Alu Elements	% of L1 Elements	% of LTR elements	% of TSSs	% of Genes
**# of Genomic Elements**	**1204532**	**10538**	**764056**	**173733**	**38956**
IgG	0.73	0.45	0.27	19.26	24.66
H3K122ac	1.5	15.83	1.85	4.39	41.44
H3K12ac	**42.92**	50.63	29.77	**76.5**	80.53
H4K16ac	**35.76**	**51.47**	**34.89**	22.86	67.99
H3K27ac	**39.63**	40.15	**33.43**	**74.44**	79.03
H3K27me3	0.36	0.06	0.21	6.76	10.14
H3K4me1	17.88	29.11	18.23	**71.55**	75.25
H3K4me3	2.86	5.1	1.12	**60.68**	60.67
H3K9me3	2.89	**47.68**	8.05	1.87	15.78

C) Percentage of reproducible peaks overlapping genomic elements						
	**Total reproducible peaks**	% of peaks at Alu	% of peaks at L1	% of peaks at LTR	% of peaks at gene TSS	% of peaks at gene body
IgG	**7966**	48.78	0.59	15.15	59.49	80.14
H3K122ac	**69622**	19.43	2.44	17.31	3.35	66.65
H3K12ac	**220291**	64.74	2.42	45.21	11.85	57.46
H4K16ac	**217029**	61.88	2.5	50.4	6.19	50.17
H3K27ac	**229431**	63.11	1.84	46.98	11.39	54.95
H3K27me3	**3820**	42.93	0.16	18.82	62.28	74.08
H3K4me1	**132905**	58.71	2.31	44.87	16.89	56.37
H3K4me3	**24013**	56.7	2.24	20.04	64.53	77.9
H3K9me3	**23612**	54.39	21.03	67.63	5.38	33.86

Supplementary Table 4. Oligos used for RT-qPCR

B-ACTIN	CAGCCATGTACGTTGCTATCCAGG	AGGTCCAGACGCAGGAT GGCATG
L1HS	CAAACACCGCATATTCTCACTCA	TGTGTCATCTAGCATTAGGTAT
L1PB	GAT GTT GGC GTG GAT GTG GT	TGG GAT TGC TGG ATC AAA TGG T
L1M	GGG GAG GTG GGG ATG GTT AA	GGT ATC CAT CCC CTC AAG CAT
L1PA 15/16	CCATTTGACCCAGCAATCCCA	CCTCCAGCTGCATCCATGTT
HERVK gag	AGC AGG TCA GGT GCC TGT AAC ATT	TGGTGCCGTAGGATTAAGTCTCCT
HERVH gag	CTTTTATTACCCAATCTGCTCCCG AYAT	TTTAGTGGTGGACAGTCTCTTTTC CARTG
MSL3	GTTATGCCACATGCCAACAT	CCAACGGGAGAGAGTGTAATCAA
NUS	TTCGGTCCTGTGGACAGCAC	CAGACGCTGTTCACAGGCTG
PEX1	ATCAGAACTTGGAAATGGAACCT	CCTGAGTCATGGAGCTTGGT
GATAD1	TCCACCAAAGGAAAAGGGAG	TGGAAACTGACTCAGGAGCTTT
USP38	CACTCCTGAAAGGACTGGCA	AACTGCAAGAGCACCAGGTC
TANC2	AAGATCCCAGAGAGAAATTTGGA	AGCAAGGTCTTCCAAGTGGG
MOXD1	TCATTGGGGTTAAGGAGATCTACAG	AGCATCCATGAAAGAAAGGTTTT
COMMD10	CAGTTGAAAAGTTCCGGCAG	TGAAGGTTAAGCTGCCATCC
CYB561	TGGTCATAGGCCTGATCTTCC	GACCTTGGTGGTGCGTTTAG
GAB1	GGATGTCGCCTTCACGTAGT	ACTTGGAAATGCTCGTGGAA
PLGRKT	GATTGCGTGGTCTCGGGAATTC	AACAATCGGGACCAGGAAGGCT
ENPP1	TGCACAGATGCCTGAACCT	ACATCCCCCAAATATTTATTCAGAT
STX7	GGAATGATGATTCATGAACAAGG	CCCTTGACAGCTGCTGATTT
RLN2	GATGCTCCTCAGACACCTAGAC	GTTGTAGCTGTGGTAATGCTGGC
SMARCA5	CCTTTGAAGATGAAACCAGGG	GCTCTGTTCTACGGTGTCGGT
	**Genotyping primers for CRISPR deletions**	
L1PA10_USP38	GGTGTGAGTGTGAATGAAACAG	GGAGCTAGGTGAAATGTACACA
L1PA7_USP38	AATATCCTACAAGAGCAGTATGGTGC	GGGGCTCAAAATTAGCAAGG
L1PA7_RLN2	CATAAGGAGGAAGGCCTCTATGC	ACTGCTTCTGATGGTATGTCCG
L1PA8_MOXD1	CCACTGCAGTGTATTAAGAGGTG	TCCAAGGCTCAGGAAATCTG

Supplementary Table 5. Details of TEs used for CRISPRi and guide RNAs

GuideRNA name	gRNA (crRNA) sequence	putative target gene
L1PA10 g1	ATGACTGAGTTTCTGAGGGG	USP38
L1PA10 g2	GGGTGGAGCAATGGCCTACT	
L1PA10 g1	CCGCAGTCTCGGTTGATCAG	TANC2
L1PA10 g2	CTGGAAACTGCCTAAGACTA	
L1PA8g1	CCTGGGAAGTGGCTAGTCTG	MOXD1
L1PA8g2	CAGGAGTTCTTACACATCAC	
L1PA7g1	CCTCTCGGTACAGTCTCTTA	COMMD10
L1PA7g2	TAGCAGCGAGAATTTCACGT	
HERVH_g1	TCAAGCTCGGGTCAAGCTCG	NUS1
HERVH_g2	ATACTTGTGGATTTAAGGTG	
HERVH_g1	ACGAGTTGGGTGCTACAGGG	PEX1/GATAD1
HERVH_g2	AAGTGGTTGATTATACTGGG	
USP38L1PA2_noAc_g1	CATGTTGGCAGTATGAAGTG	USP38
USP38L1PA2_noAc_g2	AACTGATCTAATTTAGAGGG	
USP38L1MA2_noAc_g1	GGCAATAGATCATAGAGTCT	USP38
USP38L1MA 2_noAc_g2	TGACTTATATATCTAGGTGC	
L1PA10-USP38 gRNA UP	TACCACAGCTGTTTCTGGTC	USP38
L1PA10-USP38 gRNA2 Down	TAACCCAAATAGTTATCTAC	
L1PA7-USP38 gRNA UP	CTTGAAGCAACAATCTGGTA	USP38
L1PA7-USP38 gRNA2 Down	CATCAAGTCCTCTAGATATT	
L1PA7-MOXD1gRNA UP	AGCCAACTAAGAGACTCCCC	MOXD1
L1PA7-MOXD1gRNA Down	TCGTCCAGAGAGATCTCCCA	
L1PA7- RLN2gRNA up	CAGATACTTACTTACTCCAT	RLN2
L1PA7-RLN2gRNA2 down	GTTACTACGTATGGTTAAAG	
