# Supplementary material for: H4K16ac activates the transcription of transposable elements and contributes to their cis-regulatory function
Source: Nat Struct Mol Biol. 2023 Jun 12;30(7):935–47. doi: 10.1038/s41594-023-01016-5 (PMC10352135; doi:10.1038/s41594-023-01016-5)
Supplement: Supplementary file 2 — Reporting Summary [file 41594_2023_1016_MOESM2_ESM.pdf]

nature portfolio

Corresponding author(s): Pradeepa Madapura

Last updated by author(s): 19-04-2023

Reporting Summary

Nature Portfolio wishes to improve the reproducibility of the work that we publish. This form provides structure for consistency and transparency in reporting. For further information on Nature Portfolio policies, see our Editorial Policies and the Editorial Policy Checklist.

Statistics

For all statistical analyses, confirm that the following items are present in the figure legend, table legend, main text, or Methods section.

n/a	Confirmed	
☐	☒	The exact sample size (*n*) for each experimental group/condition, given as a discrete number and unit of measurement
☐	☒	A statement on whether measurements were taken from distinct samples or whether the same sample was measured repeatedly
☐	☒	The statistical test(s) used AND whether they are one- or two-sided<br>*Only common tests should be described solely by name; describe more complex techniques in the Methods section.*
☒	☐	A description of all covariates tested
☐	☒	A description of any assumptions or corrections, such as tests of normality and adjustment for multiple comparisons
☐	☒	A full description of the statistical parameters including central tendency (e.g. means) or other basic estimates (e.g. regression coefficient) AND variation (e.g. standard deviation) or associated estimates of uncertainty (e.g. confidence intervals)
☐	☒	For null hypothesis testing, the test statistic (e.g. *F*, *t*, *r*) with confidence intervals, effect sizes, degrees of freedom and *P* value noted<br>*Give P values as exact values whenever suitable.*
☒	☐	For Bayesian analysis, information on the choice of priors and Markov chain Monte Carlo settings
☒	☐	For hierarchical and complex designs, identification of the appropriate level for tests and full reporting of outcomes
☒	☐	Estimates of effect sizes (e.g. Cohen's *d*, Pearson's *r*), indicating how they were calculated

Our web collection on statistics for biologists contains articles on many of the points above.

Software and code

Policy information about availability of computer code

Data collection	No softwares was used for data collection
Data analysis	RepeatMasker https://www.repeatmasker.org v4.0.7<br>Bowtie2 http://bowtie-bio.sourceforge.net/bowtie2/ v2.4.5<br>STAR https://github.com/alexdobin/STAR v2.7.0f<br>SAMtools http://www.htslib.org/ v1.10<br>BEDTools https://github.com/arq5x/bedtools2/ v2.28.0<br>deepTools https://github.com/deeptools/deepTools v3.5.1<br>ChromHMM http://compbio.mit.edu/ChromHMM/<br>ComplexHeatmap https://jokergoo.github.io/ComplexHeatmap/ v2.10.0<br>DESeq2 https://github.com/mikelove/DESeq2/ v1.34.0<br>Intervene https://github.com/asntech/intervene v0.6.5<br>SEACR https://github.com/FredHutch/SEACR v1.3<br>Trimmomatic v0.36<br>GraphPad Prsim v9

For manuscripts utilizing custom algorithms or software that are central to the research but not yet described in published literature, software must be made available to editors and reviewers. We strongly encourage code deposition in a community repository (e.g. GitHub). See the Nature Portfolio guidelines for submitting code & software for further information.

Data

Policy information about availability of data

All manuscripts must include a data availability statement. This statement should provide the following information, where applicable:

- Accession codes, unique identifiers, or web links for publicly available datasets
- A description of any restrictions on data availability
- For clinical datasets or third party data, please ensure that the statement adheres to our policy

All the NGS data generated in this study can be accessed at NBCI GEO under the accession ID GSE200770.
CUT&Tag, RNAseq and ATACseq datasets are deposited in the NCBI GEO datasets with following accession IDs.
CUT&Tag GSE200768
RNAseq GSE200769
ATACseq GSE200767
Details of the datasets used from other studies are mentioned in the Supplimentary Table S1.
Genome assemblies used to map the data were hg38 for human and mm10 for mouse.

Human research participants

Policy information about studies involving human research participants and Sex and Gender in Research.

Reporting on sex and gender	N/A
Population characteristics	N/A
Recruitment	N/A
Ethics oversight	N/A

Note that full information on the approval of the study protocol must also be provided in the manuscript.

Field-specific reporting

Please select the one below that is the best fit for your research. If you are not sure, read the appropriate sections before making your selection.

☒ Life sciences ☐ Behavioural & social sciences ☐ Ecological, evolutionary & environmental sciences

For a reference copy of the document with all sections, see nature.com/documents/nr-reporting-summary-flat.pdf

Life sciences study design

All studies must disclose on these points even when the disclosure is negative.

Sample size	Standard number of biological replicates and sequencing depth. For CUT&Tag, two or three biological replicates were used. For ATACseq three biological replicates were used and for RNAseq two biological replicates were used following the similar studies. For all RTqPCR experiments, 3 independent experiments were performed. For western blotting and microscopy, representative data from 2 independent experiments have been shown.
Data exclusions	No data is excluded from analyses
Replication	CUT&Tag, ATACseq and RNAseq experiments are replicated 2-3 times. For all RTqPCR experiments, 3 independent experiments were performed. For western blotting and microscopy, representative data from 2 independent experiments have been shown.
Randomization	Randomization for the samples was not required as a part of this study, as the experimentation was mainly on cell lines .
Blinding	Not applicable for this study

Reporting for specific materials, systems and methods

We require information from authors about some types of materials, experimental systems and methods used in many studies. Here, indicate whether each material, system or method listed is relevant to your study. If you are not sure if a list item applies to your research, read the appropriate section before selecting a response.

Materials & experimental systems

n/a	Involved in the study
☐	☒ Antibodies
☐	☒ Eukaryotic cell lines
☒	☐ Palaeontology and archaeology
☒	☐ Animals and other organisms
☒	☐ Clinical data
☒	☐ Dual use research of concern

Methods

n/a	Involved in the study
☐	☒ ChIP-seq
☒	☐ Flow cytometry
☒	☐ MRI-based neuroimaging

Antibodies

Antibodies used

Primary antibodies for CUT&Tag:
Normal rabbit IgG (Santa Cruz, sc-2027)
H3K27ac (Abcam, ab4729)
H4K16ac (Abcam, ab109463, Clone number EPR1004)
H3K122ac (Abcam, ab33309)
H3K4me1(Abcam, ab8895)
H3K36me3 (Abcam, ab9050)
H3K4me3 (Millipore, 07-473)
H3K27me3 (Abcam, ab192985, Clone number EPR18607)
H3K9me3 (Abcam, ab176916, Clone number EPR16601)
1 µg of primary antibodies was added to samples for CUT&Tag experiments and incubated at 4ºC overnight in a nutator.
Secondary antibody for CUT&Tag experiments:
Guinea pig α-rabbit antibody (Antibodies online, ABIN101961, 1:100 dilution)

Primary antibodies for Western blotting:
MSL3 (Merck Millipore, ABE467, 1:1000 dilution)
L1 ORF1 (Merck Millipore, MABC1152, Clone 4H1, 1:1000 dilution)
H4K16ac (Abcam, ab109463, Clone number EPR1004, 1:5000 dilution)
H3K27ac (Abcam, ab4729, 1:5000 dilution)
α-tubulin (Sigma, T9026, Clone DM1A, 1:5000 dilution)
HERV (Novus Biologicals, NB100-93579, 1:500 dilution)
Secondary antibody for Western blotting:
Goat anti-rabbit IgG H&L HRP (Abcam, ab6721, 1:3000-1:10,000 dilution)
Goat anti-mouse H&L HRP (ThermoFisher Scientific, 31430, 1:3000-1:10,000 dilution)

Validation

3 different H4K16ac antibodies from commercial vendors (Abcam, Millipore and Cell signalling technologeis) were tested for CUT&Tag. All three antibodies showed similar profile. Furthermore, western blotting, immunofluorescence (Fig 2) and CUT&Tag (Extended data Fig 4) upon MSL knockout cell lines showed significant reduction in H4K16ac CUT&Tag signal confirming the specificity of the antibodies.

Commercial antibodies, L1 ORF1, H3K27ac, H4K12ac, H3K9me3, H3K4me1, H3K4me3 antibodies are validated for western blot and ChIP. The references are available in the manufacturer's website. H3K122ac antibodies are validated in Tropberger et al Cell 2013 and Pradeepa et al 2016 Nature Genetics.
α-tubulin and HERV antibodies validated for western blot and ChIP. The references are available in the manufacturer's website.

Eukaryotic cell lines

Policy information about cell lines and Sex and Gender in Research

Cell line source(s)

H9 female hESC is procured from WiCell.
TDF iCAS9 lines are from Dr. Paola Scaffidi's lab (Crick Institute).
H1 male hESC iCAS9 lines are from Dr. Silvia Santos's lab (Crick Institute)
Prostate cancer lines (LNCaP, PC3, RWPE) are from ATCC.
HEK293T line (ATCC) is from Prof. Inderjeet Dokal's lab (Blizard Institute).
K562 (ATCC) is from Dr. Miguel Branco's lab (Blizard Institute).
SH-SY5Y (ATCC) cell line is from Dr. Elena Bochukova's lab (Blizard Institute).

Authentication

Cell lines are not authenticated

Mycoplasma contamination

Cell lines are routinely tested for mycoplasma contamination, cell lines were negative for mycoplama before performing the experiments

Commonly misidentified lines
(See ICLAC register)

No commonly misidentified cell lines used in the study

ChIP-seq

Data deposition

☒ Confirm that both raw and final processed data have been deposited in a public database such as GEO.

☒ Confirm that you have deposited or provided access to graph files (e.g. BED files) for the called peaks.

Data access links<br>*May remain private before publication.*	The data discussed in this publication have been deposited in NCBI's Gene Expression Omnibus (Pal et al., 2023) and are accessible through GEO Series accession number GSE200770 (https://www.ncbi.nlm.nih.gov/geo/query/acc.cgi?acc=GSE200770). All the datasets generated and used in this study are detailed in Supplementary Table 1.
Files in database submission	Raw Fastq files, peak files and bigwig files
Genome browser session<br>(e.g. UCSC)	HG38 genome browser session links<br><br>https://data.cyverse.org/dav-anon/iplant/home/pradeepam/H9_CutandTag_Merged_UniqueMapped/H3K27ac_merged_unq_coverage.bw<br>https://data.cyverse.org/dav-anon/iplant/home/pradeepam/H9_CutandTag_Merged_UniqueMapped/H3K27me3_merged_unq_coverage.bw<br>https://data.cyverse.org/dav-anon/iplant/home/pradeepam/H9_CutandTag_Merged_UniqueMapped/H3K36me3_unq_coverage.bw<br>https://data.cyverse.org/dav-anon/iplant/home/pradeepam/H9_CutandTag_Merged_UniqueMapped/H3K4me1_merged_unq_coverage.bw<br>https://data.cyverse.org/dav-anon/iplant/home/pradeepam/H9_CutandTag_Merged_UniqueMapped/H3K4me3_merged_unq_coverage.bw<br>https://data.cyverse.org/dav-anon/iplant/home/pradeepam/H9_CutandTag_Merged_UniqueMapped/H4K12ac_merged_unq_coverage.bw<br>https://data.cyverse.org/dav-anon/iplant/home/pradeepam/H9_CutandTag_Merged_UniqueMapped/H4K16ac_merged_unq_coverage.bw<br>https://data.cyverse.org/dav-anon/iplant/home/pradeepam/H9_CutandTag_Merged_UniqueMapped/H9WT_K122ac_merged_unq_srt_adj.bw<br>https://data.cyverse.org/dav-anon/iplant/home/pradeepam/H9_CutandTag_Merged_UniqueMapped/H9WT_K9me3_merged_unq_srt_adj.bw<br>https://data.cyverse.org/dav-anon/iplant/home/pradeepam/H9_CutandTag_Merged_UniqueMapped/Rb_IgG_merged_unq_coverage.bw

Methodology

Replicates	CUT&Tag, ATACseq and RNAseq experiments are replicated 2-3 times. Replicates agree with each other.
Sequencing depth	Sample Total read pairs (post trimming)<br>H9, H3K27ac Rep1 5267795<br>H9, H3K27ac Rep2 24283828<br>H9, H3K27ac Rep3 21041964<br>H9, H3K27me3 Rep1 1059518<br>H9, H3K27me3 Rep2 2330065<br>H9, H3K4me1 Rep1 8726469<br>H9, H3K4me1 Rep2 16756188<br>H9, H3K4me1 Rep3 35390485<br>H9, H3K4me3 Rep1 6822949<br>H9, H3K4me3 Rep2 40759484<br>H9, H3K4me3 Rep3 1452386<br>H9, H4K12ac Rep1 7805697<br>H9, H4K12ac Rep2 23829711<br>H9, H4K12ac Rep3 27318999<br>H9, H4K16ac Rep1 8831804<br>H9, H4K16ac Rep2 18212861<br>H9, H4K16ac Rep3 21797848<br>H9, H3K122ac Rep1 1434855<br>H9, H3K122ac Rep2 2802275<br>H9, H3K9me3 Rep1 16787273<br>H9, H3K9me3 Rep2 1776891<br>H9, IgG Rep1 1455306<br>H9, IgG Rep2 1000089<br>H9, IgG Rep3 10973836
Antibodies	Primary antibodies for CUT&Tag:<br>Normal rabbit IgG (Santa Cruz, sc-2027)

H3K27ac (Abcam, ab4729)
H4K16ac (Abcam, ab109463, Clone number EPR1004)
H3K122ac (Abcam, ab33309)
H3K4me1(Abcam, ab8895)
H3K36me3 (Abcam, ab9050)
H3K4me3 (Millipore, 07-473)
H3K27me3 (Abcam, ab192985, Clone number EPR18607)
H3K9me3 (Abcam, ab176916, Clone number EPR16601)
1 µg of primary antibodies was added to samples for CUT&Tag experiments and incubated at 4ºC overnight in a nutator.
Secondary antibody for CUT&Tag experiments:
Guinea pig α-rabbit antibody (Antibodies online, ABIN101961, 1:100 dilution)

Peak calling parameters

CUT&Tag peaks were called using SEACR v1.3 with relaxed (threshold 1e-6) as well as stringent (thareshold 0.01) parameters without IgG noramlisation.

Data quality

Peak detection was performed using stringent as well as relaxed mode with stringent cut-off (threshold <=1e-6).

Software

Trimmomatic v0.36 to trim the adapters
Bowtie2 http://bowtie-bio.sourceforge.net/bowtie2/ v2.4.5 for mapping to hg38 genome
SAMtools http://www.htslib.org/ v1.10 sorting and indexing bam files
SEACR v1.3 for peak calling, Repeatasker RepeatMasker v4.0.7 for repeat content analyis,
BEDTools v2.28.0 fro peak intersections,
deepTools v3.5.1 fror plotting bigwig signals across genomic landmarks
ChromHMM for genome annotation of peaks and genomic coordinates
Intervene v0.6.5 for peaks overlap across multiple datasets
RepeatMasker https://www.repeatmasker.org v4.0.7 to obtain repeat content across peaks
