# Supplementary figures and images for: H4K16ac activates the transcription of transposable elements and contributes to their cis-regulatory function
Source: Nat Struct Mol Biol. 2023 Jun 12;30(7):935–47. doi: 10.1038/s41594-023-01016-5 (PMC10352135; doi:10.1038/s41594-023-01016-5)

Uncropped blot images for Extended data Fig. 7a

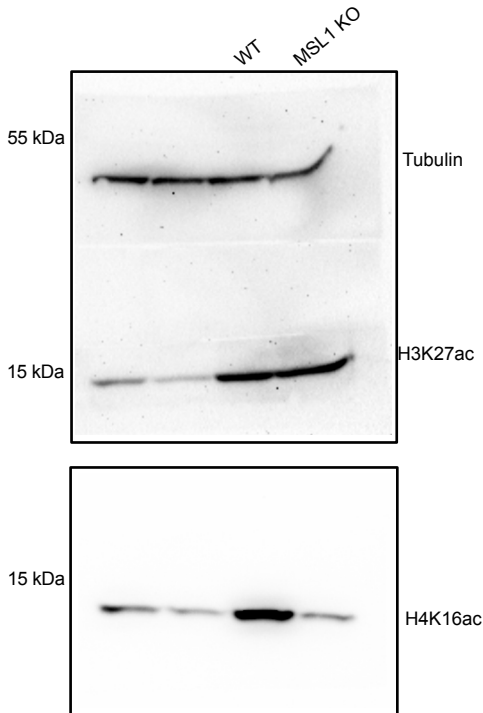

Supplement: Source Data Extended Data Fig. 7 — Uncropped western blot images for Extended Data Fig. 7a. [file 41594_2023_1016_MOESM7_ESM.pdf]
